# A Static Friction Model for Unlubricated Contact of Random Rough Surfaces at Micro/Nano Scale

**DOI:** 10.3390/mi12040368

**Published:** 2021-03-29

**Authors:** Shengguang Zhu, Liyong Ni

**Affiliations:** 1 Zhongshan Institute, University of Electronic Science and Technology of China, Zhongshan 528402, China; nily@zsc.edu.cn; 2 College of Mechanical Engineering, South China University of Technology, Guangzhou 510641, China

**Keywords:** friction, potential barrier theory, rough surfaces in contact, micro/nano scale

## Abstract

A novel static friction model for the unlubricated contact of random rough surfaces at micro/nano scale is presented. This model is based on the energy dissipation mechanism that states that changes in the potential of the surfaces in contact lead to friction. Furthermore, it employs the statistical theory of two nominally flat rough surfaces in contact, which assumes that the contact between the equivalent rough peaks and the rigid flat plane satisfies the condition of interfacial friction. Additionally, it proposes a statistical coefficient of positional correlation that represents the contact situation between the equivalent rough surface and the rigid plane. Finally, this model is compared with the static friction model established by Kogut and Etsion (KE model). The results of the proposed model agree well with those of the KE model in the fully elastic contact zone. For the calculation of dry static friction of rough surfaces in contact, previous models have mainly been based on classical contact mechanics; however, this model introduces the potential barrier theory and statistics to address this and provides a new way to calculate unlubricated friction for rough surfaces in contact.

## 1. Introduction

The accurate calculation of friction force is very complicated due to various factors, such as surface roughness, temperature, humidity, slip-weakening behavior [1,2], etc. Additionally, there are particle mechanical force, electrostatic force, van der Waals force, capillary force, etc. in the process of friction [3]. With the emergence of the surface force apparatus (SFA) and atomic force microscopy (AFM), the research emphasis of tribology is from macro to nano scale [4]. In macroscopical engineering applications, friction force is generally evaluated using classical friction laws proposed by Amontons and Coulomb et al. However, a universal and convenient formula for calculating the accurate micro/nano friction force is required because it will promote the rapid development of MEMS and NEMS technology [5].

This study focuses on dry friction force calculation research regarding nominally flat rough surfaces at the micro/nano scale. This topic has significant practical engineering application value because dry friction is a crucial factor in MEMS and NEMS applications. Although the machined surface of the component is smooth, there are several asperities on the surface at the microscopic level.

The calculation mechanism of dry friction force can be divided into two main classes [6]. One is based on classical contact mechanics. For instance, the pioneering work of Greenwood and Williamson [7], which is based on the Hertz solution for a single elastic sphere, the work of Kogut and Etsion [8], which is based on the von Mises yielding criterion for elastic-plastic contact analysis, and the works on pure elastic or pure plastic deformation of the contacting sphere [9,10]. The other class is based on various energy dissipation mechanisms that were introduced to explain friction since Tomlinson established the first phonon friction model (Independent Oscillator model) in 1929. Phononic friction was observed in an experiment conducted by Krim et al. using the QCM (quartz crystal microbalance) technique [11]. Subsequently, many phonon friction models, such as Frenkel-Kontorova (FK) model, Frenkel-Kontorova-Tomlinson (FKT) model, composite oscillator (CO) model [12], and electron models [13], etc. have been widely investigated. Additionally, the Lennard-Jones interaction potential [14] and potential barrier theory [15] were considered in friction calculations.

For dealing with rough surfaces contact of unlubricated friction, the research can be divided into two main directions [16]. One direction is based on the statistical theory, where the most widely used stochastic model is the one proposed by Greenwood and Williamson (GW model). However, other stochastic friction models, such as CEB [17], KE [18], ZMC [19], etc., are generally used for the contact between micro-rough surfaces. The other direction is based on the fractal theory, where the classical two-dimensional contact fractal model was built by Majumdar and Bhushan (MB fractal model) [20]. The statistical model is not scale-independent; however, the fractal theory can reflect on the overall information about surface topography using its own scale-independent characterization parameters (fractal dimension and fractal roughness) [21]. Nevertheless, the calculation in fractal method is more complex than that of the statistical method [22].

In this study, with the combination of energy dissipation mechanisms and statistical theory, and assuming that the contact between the equivalent asperity and rigid flat plane meets the conditions of interfacial and wear-free friction, a new dry static friction model for the contact between random micro-rough surfaces was proposed.

## 2. Modeling

### 2.1. Spherical Contact Friction Model Based on the Interfacial Potential Barrier Theory

**Figure 1:**
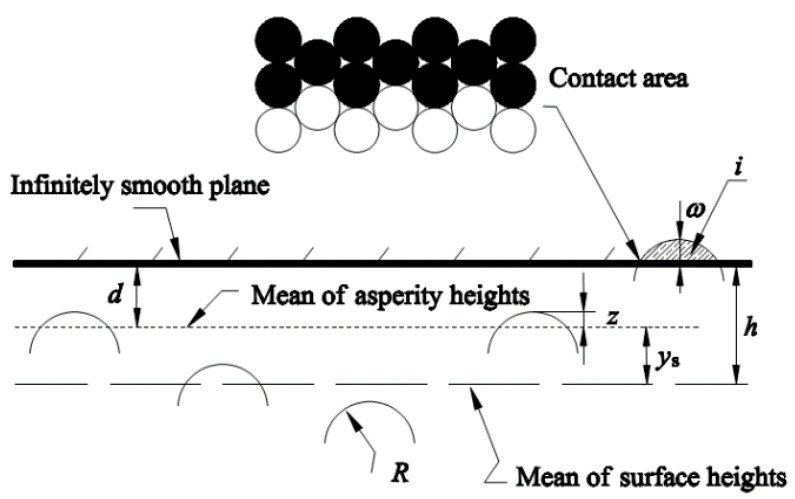
Contact model of nominally flat rough surfaces [23].

The basic assumptions of the pioneering work of Greenwood and Williamson [7] regarding the shape and statistical surface height distribution of the asperities are adopted in the present analysis. Hence, the rough surface is isotropic, all asperity heights vary randomly, there is no interaction between neighboring asperities, and there is no bulk deformation. Greenwood and Tripp [23] showed that the contact of two rough surfaces can be replaced by the contact of a single equivalent rough surface with a smooth surface, as shown in Figure 1. *R* is the uniform asperity radius of the curvature; moreover, *z* and *d* denote the asperity height and separation of the surfaces, respectively, measured from the mean of the original asperity heights. The separation *h* is measured from the reference plane defined by the mean of the original surface heights. Additionally, ω is the interference; as ω=z−d, only asperities with positive interferences are in contact.

At the micro-scale, the interface atoms come into contact because the solids are strongly volume-dependent and the surface relaxation is very small [24]. Therefore, the atoms can be regarded as densely stacked rigid balls (see the contact area of Figure 1), like the cobble stones [25]. Assuming that the contact between the equivalent asperity and the rigid flat meets the conditions of interfacial and wear-free friction, we can first analyze the static friction between a single rough peak and a rigid flat using the contact potential barrier theory.

**Figure 2:**
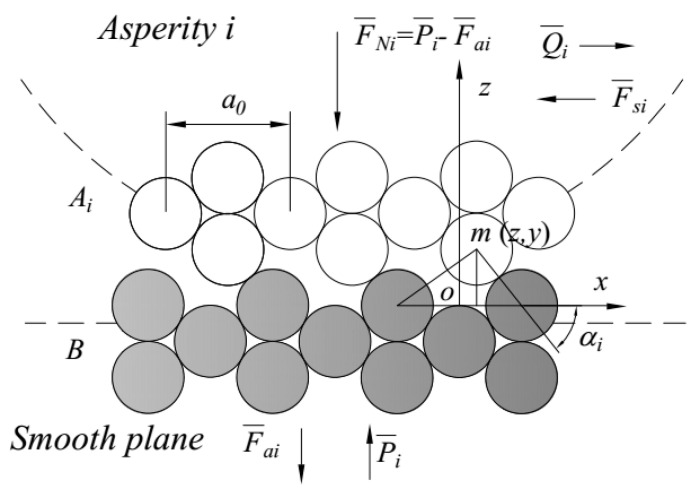
Schematic diagram of contact interface pre-displacement or initial position.

Under the action of the external normal force ${\bar{F}}_{Ni}$ and tangential force ${\bar{Q}}_{i}$, the asperity *i* slides on the smooth plane, as shown in Figure 2. The interfacial potential energy changes with the micro-relative position of the interfacial atoms. According to the interfacial contact potential barrier theory [25,26,27], the interfacial contact potential barrier directly affects the interface friction and wear properties, which can be denoted as follows:

(1) $${\bar{F}}_{s\_i}={\bar{Q}}_{i}=g(T)\cdot k_{i}\cdot \frac{\Delta U_{i}}{\Delta x_{i}}$$

where ${\bar{F}}_{s\_i}$ is the static friction of asperity *i*, g(T) is the temperature coefficient, $k_{i}$ is the position commensurate coefficient, and $\Delta U_{i}$ is the change in interfacial potential energy with the pre-displacement $\Delta x_{i}$. Lecorun assumed that objects perform a pre-displacement proportional to the external tangential force before macro-sliding; this assumption was later confirmed by Rankin et al. [28]. The tangential force corresponding to the limit pre-displacement is the maximum static friction force.

The calculation of $\Delta U_{i}$ can be based on the universal adhesion energy function proposed by Smith et al. [27] in their research on the equivalent crystal theory. By combining quantum mechanics theory with experiments, Smith et al. showed that the potential energy of the contact interface, which is composed of metal or covalent materials, similarly changes with interfacial clearance after being adjusted by the ratio parameters $E_{0}$ and *l*. The potential or adhesion energy of the contact interface of different materials can be expressed as follows:

(2) $$U(z)=U_{0}\cdot U_{*}(z_{*})$$

where $U_{0}$ is the adhesion energy when the equilibrium distance between the interfacial atoms is $z_{m}$, $U_{0}=w\cdot \bar{A}$, $\bar{A}$ is the true contact area, w is the potential energy or adhesion energy of the contact interface per unit area as follows [15].

(3) $$w=\gamma_{A}+\gamma_{B}-\gamma_{\mathrm{AB}}$$

where $\gamma_{A}$ and $\gamma_{B}$ are the surface free energy of the contact surfaces, respectively, and $\gamma_{\mathrm{AB}}$ is the interfacial free energy. For the same friction pair material, $\gamma_{A}=\gamma_{B}=\gamma$ and *γ*_AB_ ≈ 0.

In Equation (2), $U_{*}(z_{*})$ is a universal approximate function expressed by the Rydberg function [24]:

(4) $$U_{*}(z_{*})=\;-\;(1+z_{*})e^{-z_{*}}$$

where $z_{*}$ is the interface clearance after a proportional adjustment:

(5) $$z_{*}=(z-d_{m})/l$$

where *z* is the interface clearance and l is the length ratio parameter [24].

(6) $$l=\sqrt{2\gamma d/E_{\mathrm{int}}}\approx 2\sqrt{2\gamma /(12\pi E_{\mathrm{bod}}r_{\mathrm{WS}})}$$

Here, $E_{\mathrm{int}}$ is the elastic modulus of the interface, $E_{\mathrm{bod}}$ is the bulk modulus of elasticity, and $r_{\mathrm{WS}}$ is the Wigner-Seitz radius.

In the interfacial contact potential barrier period, the maximum interfacial potential energy difference of asperity *i* can be denoted as:

(7) $$\begin{array}{ll} \Delta U_{\max\_i}^{} & =U_{i}\left(z_{\max}\right)-U_{i}\left(z_{\min}\right)\\ & =U_{0}\cdot \left[-(1+z_{*})e^{-z_{*}}\right]-(-U_{0})\\ & =U_{0}\left[1-(1+z_{*})e^{-z_{*}}\right] \end{array}$$

where $U(z_{\max})$ is the interfacial potential energy (adhesion energy) at the maximum interface clearance $z_{\max}$, $U(z_{\min})$ is the interfacial potential energy at the minimum interface clearance $z_{\min}$, and $U(z_{\min})=-\Delta U_{0}$. The interface clearance of $z_{\max}$ and $z_{\min}$ can be calculated according to the crystal structure of the friction pair materials.

**Figure 3:**
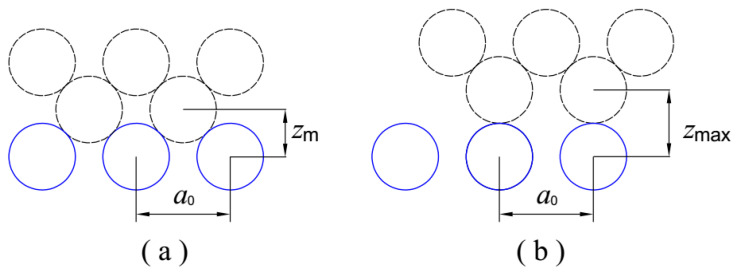
Variation in interfacial space. (**a**) Minimum space of fcc metals; (**b**) maximum space of fcc metals.

For simplicity, only one friction material is analyzed in the present work. For face-centered cubic (fcc) metals, as shown in Figure 3, the variation in the interfacial clearance in each potential cycle can be obtained by

(8) $$\delta =z_{\max}-z_{m}=\frac{\sqrt{2}}{2}a_{0}-\frac{1}{2}a_{0}\approx 0.207a_{0}$$

where $a_{0}$ is the lattice constant. Similarly, in the case of body-centered cubic (bcc) materials, the variation is calculated as follows:

(9) $$\delta =z_{\max}-z_{m}=\sqrt{{\left(\frac{\sqrt{2}}{2}a_{0}\right)}^{2}-{\left(\frac{1}{2}a_{0}\right)}^{2}}\approx 0.207a_{0}$$

Thus, we have

(10) $$z_{*}=0.207a_{0}/l$$

By substituting Equation (10) into Equation (7), then into Equation (1), and noting that $U_{0\_i}=w\cdot \bar{A_{i}}$, we can obtain the maximum static friction of asperity *i* for the same friction pair material with fcc or bcc crystal structure as follows:

(11) $${\bar{F}}_{s\max\_i}=4g(T)k_{i}\frac{\gamma {\bar{A}}_{i}}{a_{0}}\left[1-\left(1+\frac{0.207a_{0}}{l}\right)e^{-\frac{0.207a_{0}}{l}}\right]$$

### 2.2. Multi-Asperities Contact

The contact friction of rough surfaces is the statistical result of the friction of each asperity; therefore, we have:

(12) $$F_{s\max}=\sum_{i=1}^{n}{\bar{F}}_{s\max\_i}=4g(T)\frac{\gamma }{a_{0}}\left[1-\left(1+\frac{0.207a_{0}}{l}\right)e^{-\frac{0.207a_{0}}{l}}\right]\sum_{i=1}^{n}k_{i}\cdot {\bar{A}}_{i}$$

The static friction coefficient of dry rough surfaces follows the CEB friction model [17], which incorporates the three basic dry friction elements pointed by Tabor:

(13) $$\mu =\frac{Q_{\max}}{F_{N}}=\frac{F_{s\max}}{P-F_{a}}$$

where $F_{N}$ is the external normal force, and the external load is balanced by the contact load *P* and the intermolecular adhesion force $F_{s}$.

The contact of each peak is different when the rough surface contacts the rigid flat, which can be expressed by the position commensurate coefficient, *k_i_*, (see Equation (1)). 

Under a certain external normal load, the higher peaks contact the rigid flat first and the potential energy is at the lowest level if the positions of the interfacial contact atoms are fully adjusted (define *k_i_* as 1); some lower peaks also make contact, but the positions of their interface atoms are not fully adjusted (define 0 < *k_i_* < 1) and their potential energy is in an unsteady state. However, most of the rough peaks do not make contact owing to their low height (define *k_i_* as 0). For the adjustment of the position of the contact interfacial atoms involved in crystal dynamics and thermodynamics, *k_i_* can be obtained by the quantum mechanics method. However, this method is too complicated to be applied in engineering. For this reason, a statistical coefficient of positional correlation, *B* is proposed in this study and is denoted as Equation (14), and is simplified to the mean value of the ratio of the dimensionless normal load variation ($\Delta P^{*}$) to the dimensionless separation variation ($\Delta h^{*}$).

(14) $$B=\frac{\sum_{i=1}^{n}k_{i}\cdot \bar{A_{i}}}{\sum_{i=1}^{n}\bar{A_{i}}}\propto E\left(\frac{\Delta P^{*}}{\Delta h^{*}}\right)$$

For a certain interface separation between the equivalent rough surface and rigid flat, there is a contact angle $\alpha_{i}$ between the surface atoms of asperity *i* and that of the rigid flat, as shown in Figure 2. The larger the normal force, smaller the interface separation and contact angle. Moreover, *B*, which denotes the sufficiency of position adjustment of the atom, increases. Therefore, *B* is positively correlated with the normal load and negatively correlated with interface separation in the 0≤B≤1 range.

The operator on the right side of Equation (15) was chosen from the universal adhesion energy function proposed by Smith [24]. To make subsequent calculation expressions concise, we replaced this operator with the symbol D as follows:

(15) $$D=1-\left(1+\frac{0.207a_{0}}{l_{}}\right)e^{-\frac{0.207a_{{}_{0}}}{l_{}}}$$

Therefore, Equation (12) can be expressed as:

(16) $$F_{s\max}=4g(T)\frac{BD\gamma }{a_{0}}\sum_{i=1}^{n}{\bar{A}}_{i}$$

From Equation (16), the key to obtaining the maximum static friction lies in how to obtain the statistical values of the temperature coefficient, positional correlation coefficient, and real contact area. The positional correlation coefficient has been discussed above. Accordingly, the other two parameters are discussed below.

The interface atoms have their energy at a certain temperature *T*. Additionally, the required power to pull them over the potential energy is smaller than the exclusion of their energy; therefore, the temperature coefficient *g*(*T*) is introduced. According to the quantum mechanics theory, power is a mean value concerning the law of statistical distribution. Therefore, the probability of contact interfacial atoms jumping to a nearby stable location because of their energy can be calculated as follows [29]:

(17) $$\frac{dp(t)}{dt}=-f_{0}\exp\left[\frac{\Delta E_{a}(t)}{k_{B}T}\right]p(t)$$

where $f_{0}$ is the characteristic migration frequency of the friction system; $\Delta E_{a}(t)$ is the potential barrier of the interfacial atom at time *t*; *k_B_* is the Boltzmann constant, and the temperature coefficient *g*(*T*) can be estimated by:

(18) $$g(T)=\int_{0}^{a/v}p(t)dt$$

where *a* is the potential cycle and *v* is the sliding velocity. Moreover, by combining Equations (17) and (18), we can determine the temperature coefficient *g*(*T*).

During loading, the area of contact of each asperity $\bar{A}$ depends on its interference ω. Additionally, the dependence of $\bar{A}$ on ω must be determined by the asperity mode of deformation. Moreover, the total contact area *A* is obtained by summing the individual asperity contributions using a statistical model, and the same is true for the total contact load *P* and the total adhesion force $F_{a}$:

(19) $$A=\eta A_{n}\int_{d}^{\infty }\bar{A}(z-d)\phi (z)dz$$

(20) $$P=\eta A_{n}\int_{d}^{\infty }\bar{P}(z-d)\phi (z)dz$$

(21) $$F_{a}=\eta A_{n}\int_{d}^{\infty }\bar{F_{a}}(z-d)\phi (z)dz$$

where *η* is the area density of the asperities, $A_{n}$ is the nominal contact area, and ϕ(z) is the asperity height probability density function.

Using Equations (18) and (15), the above can be converted to a continuous integral form:

(22) $$F_{s\max}=4g(T)\frac{BD\gamma }{a_{0}}\eta A_{n}\int_{d}^{\infty }\bar{A}(z-d)\phi (z)dz$$

Based on the accurate finite element analysis (FEA) of an elastic-plastic single asperity contact and based on the constitutive laws appropriate to any deformation regime, Kogut and Etsion [30] developed an elastic-plastic model for rough surface contact that is general enough to accommodate material behavior. Their convenient dimensionless expressions for contact area, contact load, and adhesion force are adopted in the present work:

(23) $$A_{}^{*}=\frac{A_{}}{A_{n}}=\pi \beta \omega_{c}^{*}\left(\begin{array}{l} \int_{d^{*}}^{d^{*}+\omega_{c}^{*}}I^{1.5}+0.93\int_{d^{*}+\omega_{c}^{*}}^{d^{*}+6\omega_{c}^{*}}I^{1.136}+\\ 0.94\int_{d^{*}+6\omega_{c}^{*}}^{d^{*}+110\omega_{c}^{*}}I^{1.146}+\int_{d^{*}+110\omega_{c}^{*}}^{\infty }I^{1} \end{array}\right)$$

(24) $$P_{}^{*}=\frac{P_{}}{A_{n}H}=\frac{2}{3}\pi \beta \omega_{c}^{*}\left(\begin{array}{l} \int_{d^{*}}^{d^{*}+\omega_{c}^{*}}I^{1.5}+1.03\int_{d^{*}+\omega_{c}^{*}}^{d^{*}+6\omega_{c}^{*}}I^{1.425}+\\ 1.4\int_{d^{*}+6\omega_{c}^{*}}^{d^{*}+110\omega_{c}^{*}}I^{1.263}+\frac{3}{K}\int_{d^{*}+110\omega_{c}^{*}}^{\infty }I^{1} \end{array}\right)$$

(25) $$F_{a}^{*}=\frac{F_{a}}{A_{n}H}=2\pi \beta \theta \left(\begin{array}{l} \int_{-\infty }^{d^{*}}J_{nc}+0.98\int_{d^{*}}^{d^{*}+\omega_{c}^{*}}J_{-0.29}^{0.298}+\\ 0.79\int_{d^{*}+\omega_{c}^{*}}^{d^{*}+6\omega_{c}^{*}}J_{-0.321}^{0.356}+1.19\int_{d^{*}+6\omega_{c}^{*}}^{d^{*}+110\omega_{c}^{*}}J_{-0.332}^{0.093} \end{array}\right)$$

where the dimensionless values are denoted by *. Here, *β* is a surface roughness parameter and *H* is the hardness of the softer material. Moreover, the dimensionless critical interference $\omega_{c}^{*}$ and the dimensionless separation $d^{*}$ are normalized by the standard deviations corresponding to the surface heights *σ*. The hardness coefficient *K* is related to the Poisson ratio of the softer material; thus, K=0.454+0.41v. Additionally, *θ* is the dimensionless adhesion parameter defined as θ=Δγ/(σH), where *I^b^*, $J_{nc}$, and $J_{c}^{b}$ are general forms of the integrand.

(26) $$I^{b}={\left(\frac{z^{*}-d^{*}}{\omega_{c}^{*}}\right)}^{b}\phi^{*}(z^{*})dz^{*}$$

(27) $$J_{nc}=\frac{4}{3}\left[{\left(\frac{\varepsilon^{*}}{d^{*}-z^{*}}\right)}^{2}-0.25{\left(\frac{\varepsilon^{*}}{d^{*}-z^{*}}\right)}^{8}\right]\phi^{*}(z^{*})dz^{*}$$

(28) $$J_{c}^{b}={\left(\frac{d^{*}-z^{*}}{\omega_{c}^{*}}\right)}^{b}{\left(\frac{\varepsilon_{}^{*}}{\omega_{c}^{*}}\right)}^{b}\phi^{*}(z^{*})dz^{*}$$

Here, *ε* is the intermolecular distance and the constants *a*, *b*, and *c* are the indexes related to the four different deformation regimes [30].

The dimensionless critical interference $\omega_{c}$ is a critical parameter that marks the transition from elastic to elastic-plastic deformation and is given by

(29) $$\omega_{c}^{*}=\frac{R}{\sigma }{\left(\frac{\pi KH}{2E}\right)}^{2}$$

where *E* is the Hertz elastic modulus.

The dimensionless critical interference $\omega_{c}^{*}$ has another form of the plasticity index Ψ defined in the GW model as

(30) $$\Psi ={\left(\omega_{c}^{*}\frac{\sigma }{\sigma_{s}}\right)}^{-0.5}=\frac{2E}{\pi KH}{\left(\frac{\sigma_{s}}{R}\right)}^{0.5}$$

The ratio $\sigma_{s}/\sigma$ is defined by the surface roughness parameter *β* in the form [31]

(31) $$\frac{\sigma_{s}}{\sigma }=\sqrt{1-\frac{3.717\times 10^{-4}}{\beta^{2}}}$$

Therefore, the dimensionless maximum static friction in the present work is as follows:

(32) $$F_{s\max}^{*}=\frac{F_{s\max}}{HA_{n}}=4g(T)\frac{\gamma BDA^{*}}{Ha_{0}}$$

By substituting the dimensionless adhesion parameter θ=Δγ/(σH) into Equation (32), another form of dimensionless maximum static friction is obtained as shown below

(33) $$F_{s\max}^{*}=4g(T)\frac{\theta \sigma BDA^{*}}{a_{0}}$$

Additionally, the static friction coefficient in the dimensionless form is as follows:

(34) $$\mu =\frac{F_{s\max}^{*}}{F_{N}^{*}}=\frac{F_{s\max}^{*}}{P_{}^{*}-F_{a}^{*}}$$

## 3. Results and Discussion

To compare the proposed model to the previous KE static friction model for rough surfaces in contact, the range of plasticity indices from Ψ=0.4 to Ψ=1.3 corresponding to the steel to steel contact is selected. Furthermore, the material properties (elasticity modulus $E_{1}=E_{2}=2.06\times 10^{11}\mathrm{Pa}$, Brinell hardness $H=7.056\times 10^{9}\mathrm{Pa}$, Poisson ratio v=0.3, lattice constant $a_{0}=0.286\mathrm{nm}$, length ratio parameter l=0.56, and surface energy of adhesion $\gamma_{1}=\gamma_{2}=\gamma =2.417J\cdot m^{-2}$) are selected as an example. A constant value of K=0.577 is used corresponding to the selected Poisson ratio, and a value of β=0.04 is selected according to the finding of Greenwood and Williamson [7]. Subsequently, the ratio *σ*/*σ_s_* that is given in Equation (31) can be assumed as $\sigma /\sigma_{s}≅1$ for practical values of *β*, and the adhesion parameter *θ* is $10^{-4}\leq \theta \leq 0.01$ for metals. Using the normal temperature T=300K, the corresponding temperature coefficient g(300)≈0.6 is based on the results of the molecular dynamics calculation in reference [32]. The asperity height probability density function ϕ(z) is assumed to be Gaussian and its dimensionless form is as follows:

(35) $$\phi^{*}(z^{*})=\frac{1}{\sqrt{2\pi }}\frac{\sigma }{\sigma_{s}}\exp(-0.5{\left(\frac{\sigma }{\sigma_{s}}\right)}^{2}{\left(z^{*}\right)}^{2})$$

From Figures 2 and 3 in Refs. [30] and [10], respectively, the statistical coefficient of positional correlation *B* can be obtained by linear fitting in the range of 0.4≤Ψ≤1.3. In this case, the approximate value of *B* is 0.254. A range of practical engineering separation $0\leq h^{*}\leq 3$ and an upper limit of normal contact pressure $P^{*}\leq 0.1$ are presented below to avoid interactions between the neighborhood asperities and bulk deformation, and the relation between $h^{*}$ and $d^{*}$ is given by [33]

(36) $$y_{s}^{*}=h^{*}-d^{*}=\frac{1.5}{\sqrt{108\pi \beta }}$$

**Figure 4:**
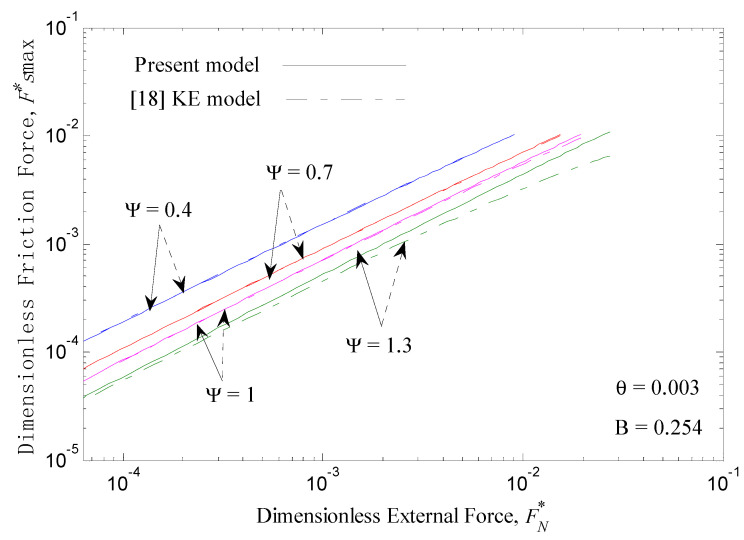
Dimensionless static friction force $F_{s\max}^{*}$ versus dimensionless external force $F_{N}^{*}$ for different plasticity indexes Ψ at θ=0.003.

Figure 4 shows the dimensionless static friction force $F_{s\max}^{*}$ versus the dimensionless external force $F_{N}^{*}$ with a relatively high adhesion parameter value (θ=0.003) for the range of plasticity indexes for fully elastic (Ψ = 0.4, 0.7) to 1st elastic-plastic (Ψ = 1, 1.3) deformations [30]. As shown, the static friction force decreases with increasing plasticity index at a given external force. A higher plasticity index means a more plastic contact or a larger standard deviation of the asperity heights. According to classical contact mechanics, the more plastic asperities there are, the lower the ability to support the tangential load, and according to the present model, a larger standard deviation of the asperity heights leads to a smaller contact area. After increasing the external force at a given plasticity index, the number of such high interference asperities increases, and various asperities that were initially non-contacting make contact; thus, the contact area increases, leading to a bigger static friction force. The trends of the static friction forces versus the external forces for different plasticity indexes in the present work are consistent with those of the KE model, which are shown in dot-dash lines in Figure 4.

**Figure 5:**
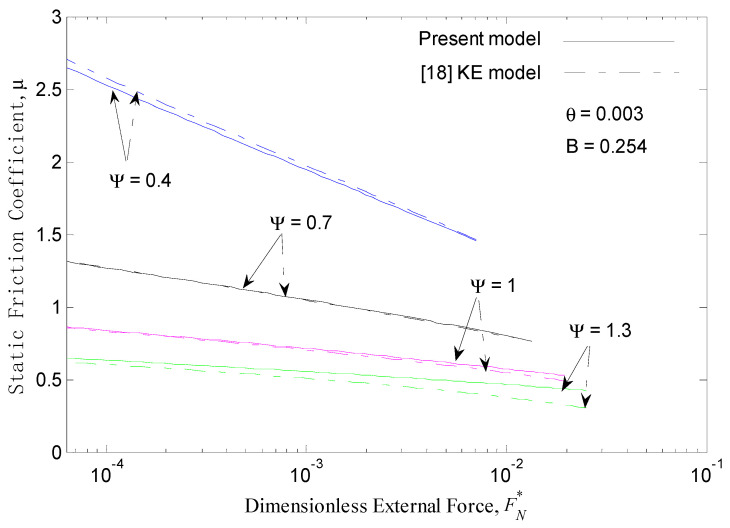
Static friction coefficient *μ* versus dimensionless external force $F_{N}^{*}$ for different plasticity indexes at θ=0.003.

Figure 5 presents the static friction coefficient *μ* versus the dimensionless external force $F_{N}^{*}$ for low and medium plasticity index values Ψ at θ=0.003. At a given plasticity index, increasing the external force decreases the static friction coefficient, and increasing the plasticity index also decreases the static friction coefficient at a given external force. The trends of the static friction coefficients vs. the external forces for different plasticity indexes in the present work are consistent with those of the KE model.

The results shown in Figure 4 and Figure 5 in dot-dash lines are from the KE static friction model [18]. The prediction results of the present model are very similar to those of the KE static friction model for the low plasticity index values (Ψ = 0.4 and 0.7) at θ=0.003. However, the results of the present work are larger than those of the KE model for the medium plasticity index values (Ψ = 1 and 1.3). Additionally, the differences between the present work and the KE model increase with an increase in the external normal force when Ψ≥1. In addition, these differences are greater when Ψ = 1.3 is used instead of Ψ = 1 at θ=0.003. Therefore, with an increase in the plasticity index, the difference in calculation results between the present work and the KE model would be greater. Because the static friction force of the model is mainly based on the contact interfacial potential barrier, as shown in Equation (16), the maximum static friction force of the present model is mainly related to the real contact area and material parameters. Additionally, it does not consider a weakening of the ability to resist tangential force when elastic-plastic or plastic deformation occurs on rough contact peaks. Following, we further analyze and discuss the change rule of the proportion of different contact deformation types to the total contact area with an increase in the plasticity index.

**Figure 6:**
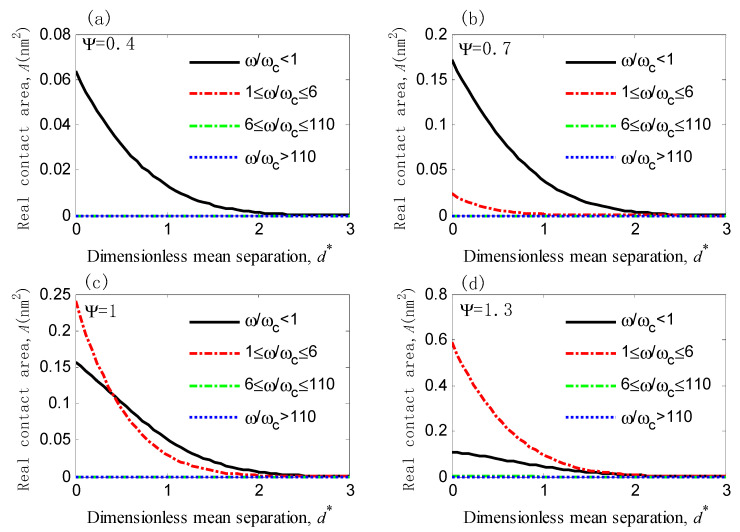
Real contact area of various deformation regimes versus dimensionless mean separation of asperities for different plasticity indexes (see Equation (23)): (**a**) Ψ = 0.4, (**b**) Ψ = 0.7, (**c**) Ψ = 1, (**d**) Ψ = 1.3.

Kogut and Etsion used the FEM to solve the problem of single asperity elastic-plastic contact and found that the entire elastic-plastic regime extends over the interference values in the $1\leq \omega /\omega_{c}\leq 110$ range with a distinct transition at $\omega /\omega_{c}=6$ [8]; consequently, the four different deformation regimes (fully elastic, first elastic-plastic, secnd elastic-plastic, and fully plastic) can be obtained. We considered four types of deformation regimes in the statistical analysis with different plasticity indexes (Ψ = 0.4, 0.7, 1, and 1.3), and the results are shown in Figure 6.

Figure 6 shows that: (a) the real contact area *A* of the fully elastic deformation is dominant for Ψ = 0.4 across the whole separation; (b) when Ψ = 0.7, the real contact area *A* of the fully elastic deformation is also the main contact area in the whole separation as the real contact area of the first elastic-plastic takes only a small part; (c) when Ψ = 1, the fully elastic deformation and the first elastic-plastic deformation coexist, and the real contact area of the first elastic-plastic deformation gradually exceeds that of the fully elastic one with a decrease in the mean separation; (d) when Ψ = 1.3, the first elastic-plastic deformation gradually occupies the dominating position with a decrease in the mean separation. Therefore, it can be concluded that the real contact area of the fully elastic deformation occupies a major part when Ψ < 1 and reduces quickly with an increase in the plasticity index. The interfacial friction potential barrier theory regards the atoms as oscillators attached by virtual springs [3] or densely stacked rigid balls [24]. Therefore, it is only applicable to frictions dominated by the fully elastic deformation. Additionally, the present work does not distinguish the ability to resist tangential force for the four types of deformations and treats them as fully elastic; hence, the prediction results of the present work are very close to those of the KE model when Ψ < 1 and greater when Ψ ≥ 1.

**Figure 7:**
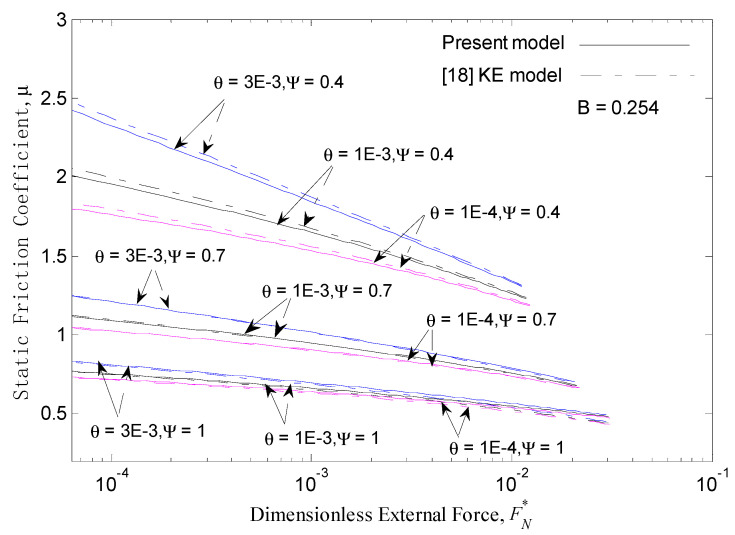
Static friction coefficient *μ* versus dimensionless external force $F_{N}^{*}$ for different plasticity index values Ψ and dimensionless adhesion parameters *θ*.

The static friction coefficient *μ* and the function of the dimensionless intermolecular adhesion force $F_{a}^{*}$ (see Equation (34)) are necessary to discuss the effect of the adhesion parameter *θ* on the static friction coefficient. The results of the present model are shown in Figure 7. Notably, the static friction coefficient decreases substantially with reducing *θ* at a given external force and a low plasticity index Ψ = 0.4. However, this effect diminishes with an increase in the external force and becomes negligibly small at the upper limit of the external force; moreover, the effect of *θ* on *μ* weakens with an increase in the plasticity index at a given external force. For the low plasticity index Ψ ≤ 1, the static friction coefficient values of the present work are very similar to those of the KE model, which are shown in dash lines.

## 4. Conclusions

A static friction model for unlubricated contact of random rough surfaces at micro/nano scale is presented to overcome the difficulty of calculating dry friction with rough surfaces. This study assumes that spherical contact meets the interfacial friction conditions, combines the potential barrier theory with the statistical theory of contact of two nominally flat rough surfaces, and proposes the statistical coefficient of positional correlation, *B* to denote the mean contact state of rough peaks. Finally, a formula for calculating the static friction related to the temperature coefficient, material property, surface energy, real contact area, and other aspects is provided.

For the calculation of the real contact area, contact load, and adhesion force, this work adopts the empirical expressions of Kogut and Etsion derived by the FEM. According to the simulation calculation results, the static friction force in this present work increases with an increase in the external normal force at a given plasticity index and decreases with an increase in the plasticity index at a given external force, which is consistent with the previous static friction models. According to the statistical analysis of the four different deformation regimes with various plasticity indexes, when Ψ < 1, the real contact area of the fully elastic deformation dominates, the results of this work are very similar to the results obtained by the KE model. However, when Ψ ≥ 1, the results of this work are greater than those of the KE model because the present work is no longer considering the weakened ability to resist tangential force for the plastic flow appearance when Ψ ≥ 1 [7].

In contrast to previous static friction models based on classical contact mechanics, the novelty of this work lies in the application of the potential barrier theory to handle friction of rough surfaces. This study provides an innovative way to predict the static friction between unlubricated rough surfaces.

The specific goal of this study is to provide a friction calculation formula for engineering applications of MEMS and NEMS. In future, we plan to conduct additional AFM related experiments to verify the present model.

## Nomenclature

$a_{0}$	lattice constant
A	real contact area
$A_{n}$	nominal contact area
B	statistical coefficient of positional correlation
$F_{a}$	external normal force
$F_{s}$	static friction force
$F_{N}$	external normal force
g(T)	temperature coefficient
h	separation based on surface heights
k	position commensurate coefficient
K	hardness factor, 0.454+0.41v
P	external normal force
R	asperity radius of curvature
z	height of an asperity measured from the mean of asperity heights
β	surface roughness parameter, ηRσ
γ	energy of adhesion
ΔU	interfacial potential difference
w	adhesion energy of contact interface
ϕ	distribution function of asperity heights
η	area density of asperities
μ	static friction coefficient
v	Poisson’s ratio of the softer material
θ	dimensionless adhesion parameter
σ	standard deviation of surface heights
$\sigma_{s}$	standard deviation of asperity heights
ω	interference
$\omega_{c}$	critical interference at the inception of plastic deformation
Ψ	plasticity index
superscript	
${}^{*}$	dimensionless

## References

[1] Albertini G., Karrer S., Grigoriu M.D., Kammer D.S. (2021). Stochastic Properties of Static Friction. J. Mech. Phys. Solids.

[2] Koji U., Rice J.R. (2003). Universal nucleation length for slip-weakening rupture instability under nonuniform fault loading. J. Geophys. Res..

[3] Krim J. (2012). Friction and energy dissipation mechanisms in adsorbed molecules and molecularly thin films. Adv. Phys..

[4] Stoyanov P., Chromik R. (2017). Scaling Effects on Materials Tribology: From Macro to Micro Scale. Materials.

[5] Kmka B., Pvs B., As B. (2018). Tribology of Silicon Surfaces: A review. Mater. Today Proc..

[6] Braun O.M., Naumovets A.G. (2006). Nanotribology: Microscopic mechanisms of friction. Surf. Sci. Rep..

[7] Greenwood J.A., Williamson J.B.P. (1966). Contact of Nominally Flat Surfaces. Proc. R. Soc. A Math. Phys. Eng. Sci..

[8] Kogut L., Etsion I. (2002). Elastic-Plastic Contact Analysis of a Sphere and a Rigid Flat. J. Appl. Mech..

[9] Cohen D., Kligerman Y., Etsion I. (2008). A Model for Contact and Static Friction of Nominally Flat Rough Surfaces under Full Stick Contact Condition. J. Tribol..

[10] Li L., Etsion I., Talke F.E. (2010). Contact Area and Static Friction of Rough Surfaces with High Plasticity Index. J. Tribol..

[11] Krim J., Solina D.H., Chiarello R. (1991). Nanotribology of a Kr monolayer: A quartz-crystal microbalance study of atomic-scale friction. Phys. Rev. Lett..

[12] Xu Z., Ping H. (2007). Study on the energy dissipation mechanism of atomic-scale friction with composite oscillator model—ScienceDirect. Wear.

[13] Liebsch A., Gonçalves S., Kiwi M. (1999). Electronic versus phononic friction of xenon on silver. Phys. Rev. B.

[14] Shengguang Z., Ping H. (2016). Calculation Model of Friction Force of Nano-Scale Rough Surface on the Basis of LJ Potential and Stochastic Processes. J. South China Univ. Technol. Nat. Sci. Ed..

[15] Xu Z., Ding L., Huang P. (2008). Interfacial potential barrier theory of friction and wear. Front. Mech. Eng. China.

[16] Gaus N., Proppe C., Zaccardi C. (2017). Modeling of dynamical systems with friction between randomly rough surfaces. Probabilistic Eng. Mech..

[17] Chang W.-R., Etsion I., Bogy D. (1988). Static friction coefficient model for metallic rough surfaces. J. Tribol..

[18] Kogut L., Etsion I. (2004). A static friction model for elastic-plastic contacting rough surfaces. J. Tribol..

[19] Zhao Y., Maietta D.M., Chang L. (2000). An Asperity Microcontact Model Incorporating the Transition from Elastic Deformation to Fully Plastic Flow. J. Tribol..

[20] Majumdar A., Bhushan B. (1991). Fractal Model of Elastic-Plastic Contact between Rough Surfaces. J. Tribol. Trans. Asme.

[21] Pan W., Li X., Wang L., Guo N., Mu J. (2017). A normal contact stiffness fractal prediction model of dry-friction rough surface and experimental verification. Eur. J. Mech. A Solids.

[22] Xiao B., Huang Q., Wang Y., Chen H. (2021). A fractal model for capillary flow through a single tortuous capillary with roughened surfaces in fibrous porous media. Fractals.

[23] Greenwood J.A., Tripp J.H. (1970). The Contact of Two Nominally Flat Rough Surfaces. Proc. Inst. Mech. Eng..

[24] James H.R., Smith J.R., Ferrante J. (1983). Universal features of bonding in metals. Phys. Rev. B.

[25] Israelachvili J.N. (1992). Adhesion, Friction and Lubrication of Molecularly Smooth Surfaces. Fundamentals of Friction: Macroscopic and Microscopic Processes.

[26] Smith J.R., Perry T., Banerjea A., Ferrante J., Bozzolo G. (1991). Equivalent crystal theory of metal and semiconductor surface and defects. Phys. Rev. B.

[27] Xu Z., Huang P. (2007). Study on Interfacial Potential Barrier Theory of Friction and Wear. Tribology.

[28] Rankin J.S. (1957). LXXIII. The elastic range of friction. Philos. Mag..

[29] Gnecco E., Bennewitz R., Gyalog T., Meyer E. (2001). Friction experiments on the nanometer scale. J. Phys. Condens. Matter.

[30] Kogut L., Etsion I. (2003). A Finite Element Based Elastic-Plastic Model for the Contact of Rough Surfaces. Tribol. Trans..

[31] McCool J.I. (1986). Predicting Microfracture in Ceramics via a Microcontact Model. J. Tribol..

[32] So M.R., Jacobsen K.W., Stoltze P. (1996). Simulations of atomic-scale sliding friction. Phys. Rev. B.

[33] Etsion I., Amit M. (1993). The Effect of Small Normal Loads on the Static Friction Coefficient for Very Smooth Surfaces. J. Tribol..

